# Use of existing systematic reviews for evidence assessments in infectious disease prevention: a comparative case study

**DOI:** 10.1186/s13643-016-0347-9

**Published:** 2016-10-11

**Authors:** Thomas Harder, Cornelius Remschmidt, Sebastian Haller, Tim Eckmanns, Ole Wichmann

**Affiliations:** 1 Immunization Unit, Robert Koch Institute, Seestrasse 10, 13353 Berlin, Germany; 2 Unit for Healthcare-Associated Infections, Surveillance of Antimicrobial Resistance and Consumption, Robert Koch Institute, Seestrasse 10, 13353 Berlin, Germany

**Keywords:** Overview of reviews, Systematic review, Case study, Infectious disease prevention, Public health

## Abstract

**Background:**

Given limited resources and time constraints, the use of existing systematic reviews (SR) for the development of evidence-based public health recommendations has become increasingly important. Recently, a five-step approach for identifying, analyzing, appraising and using existing SRs based on recent guidance by the US Agency for Healthcare Research and Quality (AHRQ) was proposed within the Project on a Framework for Rating Evidence in Public Health (PRECEPT). However, case studies are needed to test whether this approach is useful, what challenges arise and how problems can be solved.

**Methods:**

In two case studies, the five-step approach was applied to integrate existing SRs in the development of evidence-based public health recommendations. Case study A focused on the role of neonatal sepsis as a risk factor for adverse neurodevelopmental outcome. Case study B examined the efficacy, effectiveness and safety of influenza vaccination during pregnancy. For each step, we report the approach of the review team, discuss challenges and describe solutions.

**Results:**

For case study A, one existing SR was identified, while in case study B four SRs were eligible for analysis. We found that comparison of inclusion criteria alone was sufficient to judge on relevance of SRs in case study A, but not B. Although methodological quality of all identified SRs was acceptable, risk of bias assessments of individual studies included in the SRs had to be repeated in both case studies. Particular challenges appeared in case study B where multiple SRs addressed the same research question. With the help of spreadsheets comparing the characteristics of the existing SR we decided to use the most comprehensive one for our evidence synthesis and supplemented the results with those from the other SRs.

**Conclusions:**

In both case studies using the complete SR was not possible. The five-step approach provided useful and structured guidance and should be routinely applied when using existing SRs as a basis for evidence-based recommendations in public health. In situations where more than one SR has to be considered, the development of spreadsheets comparing characteristics, inclusion criteria, risk of bias, included studies and outcomes seems useful.

**Electronic supplementary material:**

The online version of this article (doi:10.1186/s13643-016-0347-9) contains supplementary material, which is available to authorized users.

## Background

During recent years, the number of published systematic reviews (SR) has increased nearly exponentially [1]. Therefore, scientists experience situations in which one or more SRs for their guideline topic already exist, leading to the question whether and how these reviews can be considered for guideline development. Moreover, limited resources and time constraints are important factors leading to considerations on the use of existing SRs, instead of performing new ones. This goes along with questions regarding the methodological quality of the existing reviews and their comprehensiveness, as well as the potential need for updating. Accordingly, the issue of using existing SRs for new reviews or guidelines has raised a number of methodological concerns [2].

Current evidence assessment frameworks try to provide guidance on how to integrate existing SRs into new reviews. The Project on a Framework for Rating Evidence in Public Health (PRECEPT), which has been initiated and funded by the European Centre for Disease Prevention and Control (ECDC), has developed methodological guidance for evaluating and grading evidence in public health, with a particular focus on infectious disease prevention and control [3]. PRECEPT explicitly recommends the use of existing SRs for evidence assessments, referring to the five-step methodology developed by the US Agency for Healthcare Research and Quality (AHRQ) and published by Robinson et al. [4]. Recently, additional guidance for this approach was published by the same group [5].

Case studies are needed to test whether this approach is useful and to identify challenges and workable solutions during the application process. Therefore, we used two recent evidence assessment projects conducted in our institute to test the applicability of the approach. These projects were related to two domains of studies comprised by PRECEPT, namely those on “risk factors” and “interventions.” In the first project, we aimed at assessing the role of neonatal sepsis as a risk factor for adverse neurodevelopmental outcome (case study A). This assessment was part of a project on the burden of healthcare-associated infections initiated and funded by ECDC. The second project comprised an assessment of the efficacy, effectiveness, and safety of influenza vaccination during pregnancy (case study B) which has been performed to support the decision-making process of the German Standing Committee on Vaccination (STIKO).

### Objectives

The objective of the study is to describe approaches, challenges, and solutions regarding the use of existing SRs for evidence assessments in infectious disease prevention and control, using a comparative case study that included two examples from different domains of research (risk factors, interventions).

## Methods

We performed a comparative case study on the use of SRs for the development of new SRs. For both case studies, systematic searches and analyses were performed by two teams of reviewers. For case study A (neonatal sepsis), SH and TH performed the searches, analyzed the data and finalized the SR, while TE acted as supervisor. For case study B (influenza vaccination), CR and TH performed the searches, analyzed the data, and finalized the SR, while OW acted as supervisor.

For the conduct of the SR of SRs, we used the approach proposed by Robinson et al. which has been incorporated in the PRECEPT framework [3]. This approach comprises five steps which guide the user through the process of identifying, analyzing, appraising, and using existing SRs. In step 1, it is suggested to locate the existing SRs, using a defined and reproducible approach. In step 2, it is proposed to assess the relevance of the identified existing reviews with regard to the new review/evidence assessment, by comparing the inclusion criteria to those of the new SR. In step 3, the assessment of the methodological quality of the identified SR(s) is suggested. Step 4 proposes four different but not mutually exclusive approaches to determine appropriate use and incorporation of existing SRs into new reviews. This includes

(i) using the included studies as a quality check for an own literature search
(ii) using the existing review to provide the body of included studies
(iii) using the data abstraction, risk of bias assessment, and/or analyses from existing SRs
(iv) using the complete existing review

**Fig. 1:**
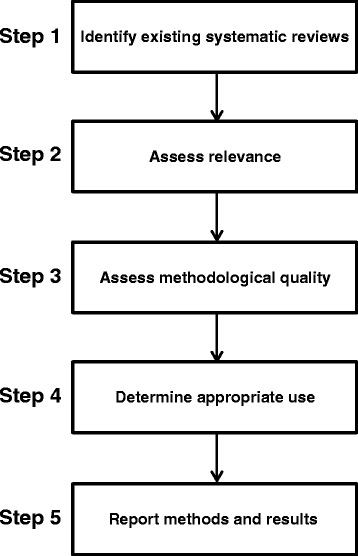
Methodological steps for identifying, assessing, and applying existing systematic reviews. Footnote: Step 4 comprises the following options: (i) using the included studies as a quality check for literature search, (ii) using the existing review to provide the body of included studies, (iii) using the data abstraction, risk of bias assessment, and/or analyses from existing systematic reviews, (iv) using the complete existing review

Step 5 suggests to report methods and results from existing SRs. The complete approach is illustrated in Fig. 1.

For each of the five steps of the approach, details on procedures performed for case studies A and B were documented. We considered challenges arising from the application of the approach for each step separately and how they were handled. Finally, a table was constructed that compares the major challenges experienced between both case studies A and B.

## Results

### Case study A: neurological sequelae of healthcare-associated sepsis in very-low-birth weight infants

#### Background

Sepsis is suspected to be a frequent cause of neurological impairment in very low birth weight (VLBW) infants. However, the risk of sequelae attributable to sepsis, particularly in the context of healthcare-associated infections, is not known. Therefore, we aimed at systematically assessing the published evidence on neurological sequelae of healthcare-associated sepsis by using existing SRs. The respective paper reporting the results of this evidence assessment has been published recently [6].

### Step 1: locate existing systematic reviews

#### Approach

To identify SRs on neurological sequelae of healthcare-associated sepsis in VLBW infants, we performed a systematic literature search in MEDLINE, EMBASE, and the Cochrane library. The search was limited to SRs published after 1 January 2000. We applied a modification of the health-evidence.ca search filter [7] which included the following terms: “Medline” OR “systematic review” OR “meta-analysis” OR “intervention.” The search led to the identification of 207 entries. After screening of titles, abstracts, and full publications, one potentially relevant SR remained for analysis (Alshaik et al. 2013 [8]) (see Appendix 1 and Additional file 1: Figure S1 for details).

#### Challenges and solutions

- To ensure that all existing SRs were identified by a transparent and reproducible approach, a systematic search was performed. A particular challenge was to develop a search strategy which was not only highly sensitive but showed a sufficient specificity to separate SRs from original studies. Therefore, we decided to apply specific search filters to narrow the results of the search.
- Although the application of search filters provided an acceptable (and plausible) number of entries, it was necessary to read the full texts from a large number of publications to decide on eligibility. In particular, even from the abstract, it was not always possible to decide whether the respective publication was a SR. Therefore, in some cases, the full text had to be assessed.

### Step 2: assess relevance of identified systematic reviews

#### Approach

To assess the relevance of the identified SR [8], we compared its inclusion criteria to a priori defined inclusion criteria of our new review. As suggested in PRECEPT, we used the PI(E)CO (population, intervention (exposure), comparison, outcome) format [3]. As shown in Table 1, this comparison revealed that differences regarding PICO were rather small. In general, the review by Alshaik et al. was more restrictive in each of the PICO criteria, with the exception of the comparator which was not explicitly defined by the authors but implicitly the same as in our review.

**Table 1:** Case study A: comparison of PI(E)CO (population, intervention (exposure), comparator, outcome) criteria between the identified systematic review (Alashaik et al. [8]) and our own new review (Haller et al. [6])

	Alshaik et al. [8]	Own new review (Haller et al. [6])
Population	Very low birth weight (VLBW) infants	Neonates (no restrictions regarding birth weight or gestational age)
Exposure	Culture-proven sepsis	Sepsis
Comparator	Not explicitly mentioned	No sepsis
Outcome	Moderate to severe neuro developmental impairment, including at least one of the following: cerebral palsy, cognitive delay (cognitive score 2SD < mean), vision loss, deafness	Any neurodevelopmental outcome

#### Challenges and solutions

- Compared to our predefined criteria, Alshaik et al. did not make any restrictions regarding study design of the included studies, although focused on cohort and case–control studies. Since we aimed at calculating risk differences in our SR, we had to limit our search to cohort studies. Therefore, we had to exclude three case–control studies and a further three studies since they did not report appropriate data for calculation of risk differences. In addition, one study did not report relevant outcomes and was therefore excluded.
- Among the 17 studies identified and reported to be eligible by Alshaik et al., there was one study that we were not able to retrieve in databases or via the Internet. After internal discussions, we decided to report this finding in the publication and proceed with the remaining studies.

### Step 3: assess quality of existing systematic reviews

#### Approach

As suggested in the guidance paper [4], we applied a commonly used tool to assess the quality of the existing SR, namely, the Assessment of Multiple Systematic Reviews (AMSTAR) tool [9]. Two independent observers assessed the quality of the SR by Alshaik et al. [8]. Four of the 11 domains of AMSTAR were found to be not adequately addressed in this SR: A list of excluded publications was not provided, no risk of bias assessment was performed and could not be used for formulating conclusions, and conflict of interest in the included studies was not investigated. Both independent researchers agreed on an AMSTAR summary score of 7/11 (Table 2).

**Table 2:** Case studies A and B: comparison of AMSTAR (Assessment of Multiple Systematic Reviews) tool domain ratings in the identified systematic reviews

AMSTAR domains^a^	Alshaik et al. [8]	Galvao et al. [14]	Jefferson et al. [15]	Fell et al. [13]	McMillan et al. [16]
Was an “a priori” design provided?	Yes	Yes	Yes	Yes	Yes
Was there duplicate study selection and data extraction?	Yes	Yes	Yes	Yes	Yes
Was a comprehensive literature search performed?	Yes	Yes	Yes	Yes	Yes
Was the status of publication used as an inclusion criterion?	Yes	Yes	Yes	No	No
Was a list of studies (include and excluded) provided?	No	Yes	Yes	Yes	Yes
Were the characteristics of the included studies provided?	Yes	Yes	Yes	Yes	Yes
Was the scientific quality of the included studies assessed and documented?	No	Yes	Yes	Yes	Yes
Was the scientific quality of the included studies used appropriately in formulating conclusions?	No	Yes	Yes	Yes	Yes
Were the methods used to combine the findings of studies appropriate?	Yes	Yes	Yes	Yes	Yes
Was the likelihood of publication bias assessed?	Yes	Yes	Yes	Yes	No
Was the conflict of interest included?	No	No	Yes	No	Yes
Summary score^b^	7	10	11	9	9

^a^According to Shea et al. [9]

^b^Maximum score: 11 (yes = 1 point)

#### Challenges and solutions

- Since a threshold for an AMSTAR score indicating “high methodological quality” has not been defined so far, uncertainties remain how to judge the results of the AMSTAR assessment. We chose to use a rather conservative approach and judged the review being of “acceptable methodological quality.” However, it remains uncertain at which AMSTAR score “threshold” researchers should refuse the use of an existing SR due to low methodological quality.
- One major AMSTAR domain that led to downgrading of the AMSTAR score was the lack of a risk of bias assessment of the included studies in the review by Alshaik et al. However, we considered this issue of minor importance since we performed our own risk of bias assessment for the new review (see below).

### Step 4: determine appropriate use and incorporate existing systematic reviews

#### Approach

Since we aimed at calculating attributable risk for each study, it was necessary to re-analyze the cohort studies included in the SR by Alshaik et al., corresponding to option (ii) in the paper by Robinson et al. [4] (see above). We performed data extraction using standardized data extraction sheets and assessed risk of bias of the original cohort studies.

#### Challenges and solutions

- In contrast to using the analysis of the existing review, re-analysis of the entire body of evidence requires much more resources. However, for our purposes, a re-analysis was necessary. At this point, the approach does not differ from the procedures used in a conventional SR regarding data extraction, checking, and data synthesis.
- We used the Newcastle Ottawa Scale to perform risk of bias assessment of all original studies included in that review [10]. Like data extraction, risk of bias assessment was performed by two independent investigators. Of the nine included studies, three had a high risk of bias, while the remaining six had a low risk of bias (for details, see [6]).

### Step 5: report methods and results from existing systematic reviews

#### Approach

Since we extracted and re-analyzed data of original studies, reporting of methods and results did not differ from the approaches used when a new SR is performed. In the respective publication, we used a table to report location of the study, birth year, population characteristics, definition of sepsis, and duration of follow-up for each included study, along with the results of the risk of bias assessment. This was accompanied by forest plots showing the risk differences from the individual studies and the pooled estimate for each outcome (cerebral palsy, vision impairment, hearing impairment, impaired neurodevelopment) (see Haller et al. [6] for details).

#### Challenges and solutions

- The approach used to report the results of the assessment was very similar to a conventional SR implicating that much more time and resources were needed, compared to a situation when data extraction and analysis from the existing SR could be used. However, using the existing results of the literature search still appears to be a time- and resource-saving strategy, compared to performing a new review.
- We decided to consider the original primary studies rather than the information reported in the SR as data source to avoid perpetuation of possible extraction errors.

### Case study B: efficacy, effectiveness and safety of influenza vaccination during pregnancy

#### Background

Influenza vaccination during pregnancy is recommended by the World Health Organization and many national health authorities to ensure protection to the pregnant woman and her newborn against influenza and associated complications [11, 12]. However, immunization during pregnancy warrants particular attention regarding adverse events. We aimed at systematically assessing the published evidence on efficacy/effectiveness and safety of vaccination against influenza during pregnancy, using existing SRs.

### Step 1: locate existing systematic reviews

#### Approach

To identify eligible SRs, we performed a systematic literature search in MEDLINE, in EMBASE, and in the Cochrane Database of Systematic Reviews. The initial search identified a total of 45 entries. After screening of titles, abstracts, and full texts, four potentially relevant SRs remained. All of them reported syntheses of data on either efficacy/effectiveness or safety of influenza vaccination during pregnancy, or both [13–16] (see Appendix 1 and Additional file 1: Figure S1 for details).

#### Challenges and solutions

- Since pregnant women are a part of the healthy adult population, we expected to find relevant information also as part of SRs on influenza vaccination in healthy adults. Therefore, we extended our search which, however, reduced specificity of the search strategy. In fact, one of the identified SRs (Jefferson et al. [15]) was a Cochrane review on vaccines for preventing influenza in healthy adults which contained a separate chapter on pregnant women and their newborns. Thereby, our case study supports the idea that some topics necessitate broadening the search to capture all relevant systematic reviews. In fact, a search limited to pregnant women only would not have identified the Cochrane review.

### Step 2: assess relevance of identified systematic reviews

#### Approach

As in case study A, we started to assess the relevance of the identified SRs by comparing their inclusion criteria to those of our own review, using the PICO format. Generally, comparison of PICOs revealed only small differences between the existing SRs, with one notable exception (Table 3). The SR by Fell et al. [13] focused on preterm birth and fetal death but did neither investigate maternal outcomes nor those related to the efficacy or effectiveness of vaccination in preventing influenza in the infant.

**Table 3:** Case study B: comparison of PICO (population, intervention, comparator, outcome) criteria between the existing systematic reviews (Galvao et al. [14], Jefferson et al. [15], Fell et al. [13], and McMillan et al. [16]) and our own new review

	Galvao et al. [14]	Jefferson et al. [15]	Fell et al. [13]	McMillan et al. [16]	Own new review
Population	Pregnant women and their infants	Pregnant women and their newborns	Pregnant women and their infants	Pregnant women, their fetuses and infants up to 6 months of age	Pregnant women and their infants
Intervention	Vaccination against influenza	Live attenuated or inactivated vaccines	Vaccination against influenza	Inactivated influenza vaccination	Vaccination against seasonal influenza
Comparator	Placebo or other vaccines or no vaccination	Placebo or no vaccination	No vaccination	No vaccination	Placebo or no vaccination
Outcome	Influenza-related outcomes in mother or infant	Symptomatic influenza and influenza-like illness; maternal and pregnancy outcomes; neonatal outcomes: congenital malformations, neonatal death	Preterm birth, early fetal death, late fetal death	Influenza, influenza-like illness, for pregnant women: adverse events and serious adverse events; for the fetus: spontaneous abortion, fetal death, premature birth, low birth weight, small for gestational age, congenital malformation	Laboratory-confirmed influenza in mother and/or infant; any severe adverse event in mother or infant

#### Challenges and solutions

- A more detailed analysis revealed that the comparison of PICOs alone would not suffice to assess the relevance of the identified SRs. In particular, by screening the SRs, we detected considerable differences regarding study designs of the included studies, numbers of included studies, inclusion of pandemic and/or seasonal influenza vaccines, etc. Therefore, we decided to construct an additional spreadsheet for comparison of characteristics of the SRs (Table 4). These comparisons revealed differences with regard to the study designs of the included studies defined a-priori. While the Cochrane review [15] and the SR by McMillan et al. [16] did not make any restrictions regarding the design of the studies to be included, Galvao et al. [14] decided to include only RCTs and cohort studies, whereas Fell et al. [13] in addition included cross-sectional and case–control studies. Three of the SRs included studies concerning seasonal as well as pandemic influenza vaccines, while the remaining SR (Galvao et al. [14]) focused on vaccination against seasonal influenza only. In three SRs, a meta-analysis was performed. Remarkably, considerable differences between the SRs were observed with regard to the number of included studies which ranged between 8 and 46.

**Table 4:** Case study B: comparison of additional characteristics between the existing systematic reviews (Galvao et al. [14], Jefferson et al. [15], Fell et al. [13], and McMillan et al. [16]) and our own new review

	Galvao et al. [14]	Jefferson et al. [15]	Fell et al. [13]	McMillan et al. [16]	Own new review
Study designs	RCTs, cohort studies	All study designs	RCTs, cohort, cross-sectional, case–control studies	All study designs	All study designs
Period covered	Until 09/2013	Until 05/2013	Until 05/2013	Until 03/2014	Until 03/2014
Seasonal	Yes	Yes	Yes	Yes	Yes
Pandemic	No	Yes	Yes	Yes	No
Meta-analysis	No	Yes	Yes	Yes	No
No. of included studies	8	21	27	46	20
Risk of bias tool(s) used for RCTs	Cochrane risk of bias tool	Cochrane risk of bias tool	NA	JBI-MASTARI	Cochrane risk of bias tool
Risk of bias tool(s) used for observational studies	NR	NOS	NOS; DBC	JBI-MASTARI	CASP
Results of risk of bias assessment	NR	10× high risk of bias; 11× unclear risk of bias	NOS: median 8.5 (of 9) DBC: median 25 (of 31)	Moderate to high quality	9× high risk of bias; 9× low risk of bias; 2× unclear risk of bias

*CASP* Critical Appraisal Skills Program, *DBC* Downs and Black Checklist, *NA* not applicable, *NOS* Newcastle-Ottawa-Scale, *NR* not reported
- Furthermore, the identified SRs differed considerably regarding included maternal and infant outcomes. To assess these differences in detail, we constructed a further table comparing the included outcomes with respect to the original studies where they were reported (Table 5). This comparison revealed that a total of 14 outcomes were reported in the four systematic reviews. The most comprehensive data base was provided by the SR by McMillan et al. [16], while the SR by Fell et al. [13], due to its inclusion criteria, reported the smallest number of outcomes.

**Table 5:** Case study B: comparison of included primary studies and outcomes in the existing systematic reviews (Galvao et al. [14], Jefferson et al. [15], Fell et al. [13], and McMillan et al. [16])

Maternal outcomes					
Outcome	Primary study	Galvao et al. [14]	Jefferson et al. [15]	Fell et al. [13]	McMillan et al. [16]
Laboratory-confirmed influenza	Thompson et al. [30]	No (unclear)	No (date)	No (criteria)	Yes
	Zaman et al. [31]	No (unclear)	No (unclear)	No (criteria)	Yes
Influenza-like illness	Black et al. [32]	No (unclear)	Yes	No (criteria)	Yes
	Hulka [33]	Yes	Yes	No (criteria)	No (unclear)
	Munoz et al. [34]	No (unclear)	No (unclear)	No (criteria)	Yes
	Zaman et al. [31]	Yes	No (unclear)	No (criteria)	No (unclear)
Local adverse events	Hulka [33]	No (criteria)	No (unclear)	No (criteria)	Yes
	Yeager et al. [35]	No (criteria)	No (unclear)	No (criteria)	Yes
	Zaman et al. [31]	No (criteria)	No (unclear)	No (criteria)	Yes
Systemic adverse events	Englund et al. [36]	No (criteria)	No (unclear)	No (criteria)	Yes
	Hulka [33]	No (criteria)	No (unclear)	No (criteria)	Yes
	Lin et al. [37]	No (criteria)	No (unclear)	No (criteria)	Yes
	Yeager et al. [35]	No (criteria)	No (unclear)	No (criteria)	Yes
	Zaman et al. [31]	No (criteria)	No (unclear)	No (criteria)	Yes
Serious adverse events	Munoz et al. [34]	No (criteria)	No (unclear)	No (criteria)	Yes
	Nordin et al. [38]	No (criteria)	Yes	No (criteria)	Yes
Preeclampsia	Munoz et al. [34]	No (criteria)	No (unclear)	No (criteria)	Yes
Infant outcomes					
Lab-confirmed influenza	Benowitz et al. [39]	No (criteria)	Yes	No (criteria)	Yes
	Eick et al. [40]	Yes	Yes	No (criteria)	Yes
	Poeling et al. [41]	No (criteria)	Yes	No (criteria)	Yes
	Zaman et al. [31]	Yes	No (unclear)	No (criteria)	Yes
Influenza-like illness	Black et al. [32]	No (unclear)	Yes	No (criteria)	Yes
	Eick et al. [40]	No (unclear)	Yes	No (criteria)	Yes
	France et al. [42]	No (unclear)	Yes	No (criteria)	No (unclear)
	Munoz et al. [34]	No (unclear)	No (unclear)	No (criteria)	Yes
	Zaman et al. [31]	Yes	No (unclear)	No (criteria)	Yes
Premature birth (<37 weeks)	Black et al. [32]	Yes	Yes	Yes	Yes
	Chambers et al. [43]	No (unclear)	No (date)	Yes	Yes
	Dodds et al. [44]	No (unclear)	No (unclear)	Yes	No (unclear)
	Legge et al. [45]	No (date)	No (date)	Yes	No (unclear)
	Louik et al. [46]	No (date)	No (unclear)	Yes	No (unclear)
	Munoz et al. [34]	Yes	Yes	Yes	Yes
	Omer et al. [47]	Yes	Yes	Yes	Yes
	Sheffield et al. [48]	Yes	Yes	Yes	Yes
	Steinhoff et al. [49]	No (unclear)	No (unclear)	Yes	No (unclear)
	Zaman et al. [31]	Yes	No (unclear)	No (unclear)	No (unclear)
Fetal death (>500 g)	Sheffield et al. [48]	Yes	Yes	Yes	Yes
Spontaneous abortion	Irving et al. [50]	No (criteria)	No (date)	No (criteria)	Yes
Congenital malformation	Munoz et al. [34]	No (criteria)	Yes	No (criteria)	No (unclear)
	Sheffield et al. [48]	No (criteria)	Yes	No (criteria)	Yes
Small for gestational age	Omer et al. [47]	Yes	No (unclear)	No (criteria)	Yes
	Sheffield et al. [48]	Yes	No (unclear)	No (criteria)	Yes
	Zaman et al. [31]	Yes	No (unclear)	No (criteria)	No (unclear)
Neonatal death	Sheffield et al. [48]	Yes	Yes	No (criteria)	No (unclear)

No, no included (with reasons for exclusion in parenthesis: date = published after search date of the SR; criteria = inclusion criteria of the SR not met; unclear = reason for exclusion from the SR unclear); Yes, included

### Step 3: assess methodological quality of existing systematic reviews

#### Approach

As shown in Table 2, all four potentially relevant reviews received high AMSTAR summary scores, ranging between 9/11 and 11/11 points. Therefore, we concluded that regarding methodological quality, the SRs were nearly equal. Differences were observed regarding single domains: Two SRs did not consider “status of publication” adequately, one did not investigate publication bias, and only two SRs considered conflict of interest in the included studies.

#### Challenges and solutions

- As discussed in case study A, it is unclear what “high methodological quality” means in terms of the AMSTAR summary scores. Furthermore, empirical studies are missing which investigate whether or not a SR with an AMSTAR summary score of 11 is superior to one with an AMSTAR score of 9. Rather, it appears to be important to consider the domains in which differences were observed. We judged all four SRs as being of adequate methodological quality.

### Step 4: determine appropriate use and incorporate existing systematic reviews

#### Approach

Since in step 2 we had identified the SR by McMillan et al. [16] as the most comprehensive one, we decided to use this SR as the base for our evidence synthesis. In those cases where single studies or outcomes were missing from the review by McMillan et al., we supplemented the results with those from the other SR, using the spreadsheet shown in Table 5 as guidance.

#### Challenges and solutions

- As show in Table 4, we observed that in the four SRs a total of four different risk of bias tools were applied to assess internal validity of RCTs and observational studies. We therefore decided to re-analyze risk of bias in the primary original studies, using the Cochrane risk of bias tool for randomized controlled trials [17] and the Newcastle Ottawa Scale for cohort studies [10]. Like data extraction, risk of bias assessment was performed by two independent investigators (for results, see Table 4).

### Step 5: report methods and results from existing systematic reviews

#### Approach

Using the approach described above, we performed a new evidence synthesis for all outcomes reported in the 20 original studies contributing to the four SRs. We aggregated the study estimates in tables, separately for RCTs and observational studies.

#### Challenges and solutions

- Data from the original studies were very heterogeneous regarding study populations, vaccines used, and outcome definitions. Therefore, although meta-analyses were performed in three of the SRs, we decided not to perform a quantitative data synthesis but to report the non-aggregated study results in tables.

### Comparison of major challenges in case studies A and B

In Table 6, we summarized the major challenges and solutions along steps 1 to 5 for both case studies. Obviously, the challenges and respective solutions at each of the five steps differed between the two case studies. Only in step 3 (assessing quality), similar challenges occurred with regard to the interpretation of the AMSTAR score. Many challenges in case study A were related to the need of performing a completely new data extraction and analysis. In case study B, the majority of challenges was caused by partial overlap of the studies included in the different SRs and the resulting question how to deal with this situation.

**Table 6:** Comparison of steps 1–5: major challenges and solutions in case studies A and B

Step number	Description	Case study A: neurological sequelae of neonatal sepsis		Case study B: influenza vaccination during pregnancy	
		Challenges	Solutions	Challenges	Solutions
1	Locate existing systematic reviews	Need to restrict search results to SRs	Use of search filters	Relevant SRs can be part of overarching SRs	Widening of the search strategy to more unspecific topics (e.g., “healthy adults”)
2	Assess relevance of the existing reviews	Non-retrievable references in relevant SR	Reporting of this finding in the new SR	PICO alone not sufficient to assess relevance	Construction of additional spreadsheets comparing characteristics of SRs and included individual studies
3	Assess quality of existing reviews	Value of AMSTAR summary score not known	AMSTAR summary score of 7/11 reported and judged as “acceptable”	Value of AMSTAR summary score not known	AMSTAR summary scores of 9/11–11/11 reported and judged as “appropriate”
4	Determine appropriate use and incorporate existing reviews	Numbers and risk of bias not reported in SR	New data extraction from individual studies and risk of bias assessment	Different risk of bias tools used in the relevant SRs	New risk of bias assessment with a defined tool
5	Report methods and results from using existing reviews	Aggregated data and quantitative synthesis from SR not appropriate	Summary of data from the individual studies and conduct of new meta-analysis	Heterogeneity of study characteristics and results	Narrative data summary, results of individual studies in tables, no meta-analysis

*SR* systematic review

## Discussion

Major aim of our work described here was to test the application of the AHQR approach in the context of using systematic reviews for developing evidence-based recommendations in the field of infectious disease prevention and control. Importantly, this might also include research questions which do not come from the field of comparative effectiveness but include topics like risk factors or incidence. Nevertheless, we conclude that the approach is applicable to these scenarios and provides excellent guidance for the use of systematic reviews also in this context.

The use of existing SRs for the development of new SRs has been suggested to be an efficient way to develop evidence-based recommendations in public health, offering a time- and resource-saving alternative to the conduct of a new SR. Our comparative case study shows that applying the five-step approach by Robinson et al. [4] which is integrated in the PRECEPT evidence assessment framework [3] leads to a transparent evidence assessment based on existing SRs. However, depending on the quantity and quality of existing SRs for the given research question, different challenges have to be managed during the evidence assessment process. Furthermore, we experienced that in cases when extensive re-analysis was needed because the existing SRs did not report appropriate data from the primary studies, the advantages of using existing SRs over performing a new SR are likely to be small.

In our experience in conducting systematic reviews “from scratch,” 1/3 of time has to be spent for literature search, 1/3 for data extraction, and 1/3 for data analysis and interpretation of the data, respectively. We think that it is worth using existing reviews, if at least one of these three “steps” can be omitted. We estimated that a full systematic review takes six to 24 months, depending on complexity of question, number of relevant outcomes, relevant publication dates, etc. In our experience, by using existing systematic reviews instead of starting a new one, this process can be shortened by about one to two thirds, depending on topic, comprehensiveness, and quality of the existing reviews.

Overviews of SR are a new approach of evidence synthesis which becomes increasingly popular [18]. However, it has been recognized earlier that a number of methodological challenges exist related to these approaches [19]. So-called umbrella reviews can be considered to be a specific form of SRs of SRs by focusing on meta-synthesis of the results of comparable meta-analyses [20]. However, our case study illustrates that it might be of equal importance to develop methodological guidance for those cases where detailed comparison and dissection of the existing SRs rather than synthesis are needed. For the development of the PRECEPT framework, we decided to use the approach proposed by Robinson et al. [4] which has recently been further elaborated by the same group [5].

Our case studies A and B showed large differences regarding the evidence base. While case study A had to build on only one SR, for case study B four SRs were identified. At first look, these SRs appeared very similar regarding methodological quality and thematic relevance. However, further analysis revealed several challenges resulting from differences, e.g., in included studies, considered outcomes and risk of bias assessments. All reviews included in both case studies had very broad PICO questions, particularly with regard to the outcomes. The systematic reviews on influenza vaccination during pregnancy for example did not focus on one or two outcomes, but used groups of outcomes such as “adverse events during pregnancy” which were presented in an aggregated as well as non-aggregated way. Comparing the systematic reviews on influenza vaccination during pregnancy we realized that the authors transformed the data differently, e.g., regarding outcomes such as “fetal death,” “stillbirth,” “abortion,” or “intrauterine death.”

Interestingly, already 20 years ago, Jadad et al. discussed problems which might arise when SRs on the same topic come to discordant results or conclusions [21]. In our study, one particular challenge arose from the fact that the four SRs had partially overlapping studies. A recent study by Siontis et al. suggested that this problem is not uncommon. Of 73 meta-analyses analyzed in their article, 67 % had at least one other overlapping meta-analysis [22]. Siontis et al. proposed three possible solutions for such a scenario: (i) to select the most appropriate SR, (ii) to perform a new review, or (iii) to synthesize the results of the reviews. Similar challenges in analyzing and using existing SRs with overlapping topics have been recently described by Bolland and Grey [23]. Like in our study, these authors observed important differences in trial selection between seven meta-analyses on vitamin D supplements. Other researchers have developed graphical tools to assist in the assessment of appropriateness of each meta-analysis for a given research question [24]. In our study, we decided to apply the concept of comparative spreadsheets suggested by Whitlock et al. [2] to compare certain features of the included systematic reviews to assess their value for a new evidence synthesis. We thereby identified the most comprehensive review [16] and supplemented this study base with the results of the other three SRs. However, it has to be considered that our approach of selecting the most comprehensive review and supplementing it with studies identified in other reviews does not always work. The most comprehensive review may not be of the highest quality, it ignores the possibility that the selection criteria of the review authors were not entirely transparent, it does not eliminate the need for risk of bias assessment, and it still might require evidence assessments to be done. Furthermore, AMSTAR does not completely separate assessment of bias from reporting quality. It has to be investigated whether the recently developed ROBIS tool [25] performs better in capturing risk of bias in systematic reviews.

In step 4 of the approach, it is suggested to either use the results of the risk of bias assessments from the existing SRs for the new review or to perform a new assessment. In case study A, the latter approach was ultimately necessary since the existing review did not report risk of bias in the included studies. On the contrary, in case study B, all included SRs reported their own risk of bias assessments. However, these were based on four different risk of bias tools. In all SRs, assessments of RCTs were performed by using the Cochrane risk of bias tool [17], whereas for observational studies either the Newcastle Ottawa Scale [10], the Downs and Black Checklist [26], or the JBI-MASTARI tool [16] was used. The authors of these SRs came to differing conclusions regarding the methodological quality of the included original studies. Although a comparison of risk of bias assessments between the reviews would be interesting, reporting quality of these results showed large differences, making a direct comparison impossible: The systematic review by Fell et al. reported numerical quality scores per study, but not per outcome, using the Newcastle Ottawa Scale and the Downs-Black Scale. In the Methods section of the review by Galvao et al., it was declared that the Cochrane risk of bias tool was used, but the authors did not report the results of this assessment. In the systematic review by McMillan et al., the JBI-MASTARI was used, but the results of this assessment were reported in aggregated form only, rather than for individual studies and outcomes. However, one has to take into account that even the use of identical risk of bias tools can lead to different judgements on methodological quality by different authors, since it has been demonstrated that many risk of bias tools show a considerable degree of inter-rater variability [10, 27]. Taking all these uncertainties into account, we decided to perform a new risk of bias assessment for our review, and compared the results to those by the other investigators.

Remarkably, quality of the evidence was not assessed in the original systematic review by Alshaik et al. which served as the base for our case study A. Regarding the systematic reviews used in case study B, only one (Galvao et al.) of four reviews reported a GRADE quality assessment. However, due to differences in the number of included studies, it is difficult to compare the results of the assessment made by those authors to our GRADE assessments. Furthermore, updating of existing systematic review constitutes an important step which should be considered in every situation when the use of existing reviews is discussed as an approach in the development of evidence assessments. Importantly, methods have been developed to evaluate the need for updating, such as the surveillance system proposed by AHRQ [28].

## Conclusions

Our comparative case study supports the applicability of the five-step approach [4] included in the PRECEPT framework for the use of existing SRs. However, efforts and challenges depend on the quality and quantity of the evidence in the SRs. Exclusion of non-systematic (or narrative) reviews constitutes a particular challenge since they can often not been identified without reading the full text of the respective article. Important progress in literature searches could be made if this kind of reviews would be adequately termed and indexed in electronic databases. Adherences of all SR authors to the PRISMA guideline [29] would strongly improve the quality of published reviews. Authors of systematic reviews should provide a database (e.g., an Endnote file) alongside with the paper to support replication, re-analysis, and updating of the respective systematic reviews. In situations where more than one SR has to be considered, the development of spreadsheets comparing characteristics, inclusion criteria, risk of bias, included studies, and outcomes can support the development of evidence-based guidance. For such situations, we used our experiences documented in this paper to develop a recommendation on how to proceed (Table 7). Furthermore, our study identified a number of open questions which should be addressed in future studies, such as

**Table 7:** Recommendation: how to proceed with overlapping systematic reviews on the same topic

1)	Prepare a matrix of studies, exposures and/or outcomes
2)	Select the most appropriate/comprehensive systematic review as “base”
3)	Supplement the “base” with studies included in the other systematic reviews
4)	Compare extractions
5)	Check extractions
6)	Assess risk of bias
7)	Perform tabular/narrative synthesis
8)	Perform meta-analysis, if useful and needed

- What is a “good” AMSTAR score?
- How to deal with overlapping studies when using existing systematic reviews?
- When is it more efficient to perform a new systematic review (in terms of time and resources) than to try to use an existing one?

The use of a complete SR is often not possible and intensive re-analyses are necessary. Still, even when such reanalyses are needed, the use of existing SRs is a promising alternative to the conduct of a new SR and might support fast and efficient development of evidence-based recommendations in public health.

## Appendix

### Appendix 1

Search strategy for case study A (Databases searched: MEDLINE, EMBASE, and the Cochrane Central Register of Controlled Trials, date of last search: July 2, 2014).

#1 “outcome”

#2 “follow-up”

#3 “sequel*”

#4 “consequence”

#5 “death”

#6 “cerebral palsy”

#7 “retinopathy”

#8 “necrotizing enterocolitis”

#9 “bronchopulmonary dysplasia”

#10 “neurodevelopmental impairment”

#11 “periventricular leukomalacia”

#12 “intraventricular haemorrhage”

#13 “neonat*”

#14 “newborn”

#15 “sepsis”

#16 (#1 OR #2 OR #3 OR #4 OR #5 OR #6 OR #7 OR #8 OR #9 OR #10 OR #11 OR #12) AND (#13 OR #14) AND #15

Filters: publication date from 2000/01/01; humans; “Medline” OR “systematic review” OR “meta-analysis” OR “intervention”

Search strategy for case study B. (Databases searched: MEDLINE, EMBASE and the Cochrane Database of Systematic Reviews, date of last search: October 8, 2014)

#1 “pregnan*”

#2 “gestation*”

#3 “influenza”

#4 “vaccin*”

#5 “immuni*”

#6 “systematic review”

#7 “meta-analysis”

#8 (#1 OR #2)

#9 (#4 OR #5)

#10 (#6 OR #7)

#11 3 AND 8 AND 9 AND 10

Filters: −

## Additional file

Additional file 1: Case study A: flow chart for the systematic literature search and study selection. (DOCX 87 kb)

## Abbreviations

AHRQ: Agency for Healthcare Research and Quality
AMSTAR: Assessment of Multiple Systematic Reviews
PRECEPT: Project on a Framework for Rating Evidence in Public Health
SR: Systematic review
