# Supplementary material for: Use of existing systematic reviews for evidence assessments in infectious disease prevention: a comparative case study
Source: Syst Rev. 2016 Oct 11;5:171. doi: 10.1186/s13643-016-0347-9 (PMC5057474; doi:10.1186/s13643-016-0347-9)
Supplement: Additional file 1: — Case study A: flow chart for the systematic literature search and study selection. (DOCX 87 kb) [file 13643_2016_347_MOESM1_ESM.docx]

**Appendix 2**

**Case study A**: Flow chart for the systematic literature search and study selection.

**
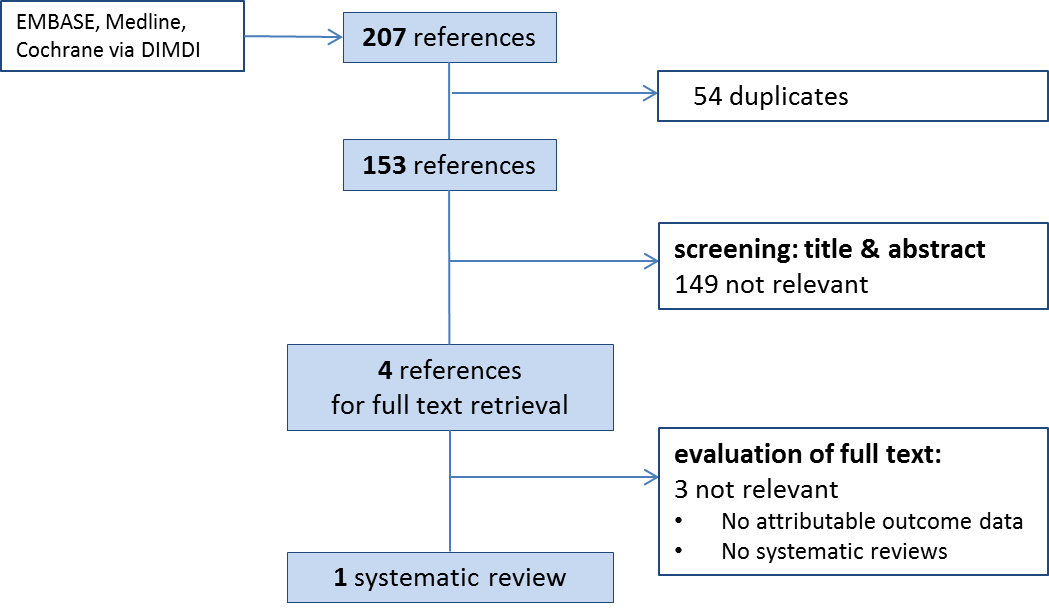
**

**Case study B**: Flow chart for the systematic literature search and study selection.

**
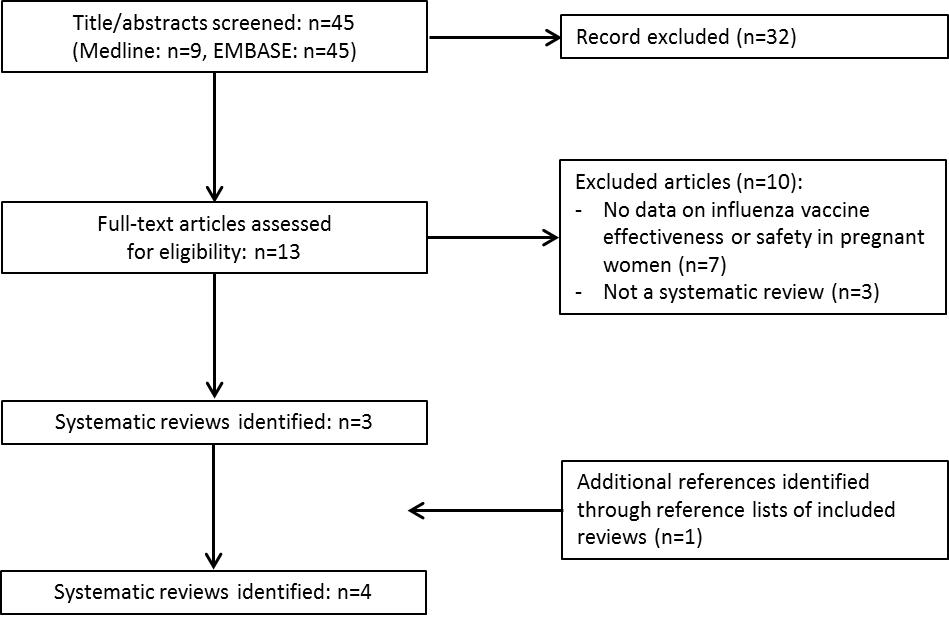
**
